# Predictors of Clinical Success in Resin Infiltration for MIH Opacities

**DOI:** 10.3390/jcm15010124

**Published:** 2025-12-24

**Authors:** María Dolores Casaña-Ruiz, Mª Angeles Velló-Ribes, Montserrat Catalá-Pizarro

**Affiliations:** Department of Stomatology, Faculty of Medicine and Dentistry, University of Valencia, 46010 Valencia, Spain; m.angeles.vello@uv.es (M.A.V.-R.); montserrat.catala@uv.es (M.C.-P.)

**Keywords:** molar incisor hypomineralization, MIH, resin infiltration, pediatric dentistry, predictability

## Abstract

**Background/Objectives**: Enamel defects in molar-incisor hypomineralization (MIH) have a multifactorial etiology involving environmental, systemic, and genetic factors. These alterations represent an aesthetic and emotional challenge, especially in anterior teeth. Resin infiltration has emerged as a minimally invasive treatment for MIH opacities, though outcome predictability remains limited. This study aims to analyze the baseline characteristics of MIH enamel defects and identify specific patterns that may predict clinical outcomes. **Methods**: This was a single-arm, prospective, observational clinical study with a six-month follow-up, with a total of 101 MIH-affected teeth treated with Icon^®^ resin infiltration. Opacities were analyzed using CIELAB color parameters (Lab*), including luminance, lesion extent, affected tooth type, opacity location, and patient age. Treatment success was assessed using simple linear regression models with generalized estimating equations, which were based on different covariates. Clinical success was defined as the combined achievement of a significant reduction in ΔE, a decrease in L* indicating reduced opacity brightness, and a reduction in the relative surface area of the lesion at six months. **Results**: White opacities showed greater reduction after infiltration than yellow and brown ones (*p* < 0.029). Larger lesions exhibited greater improvement (*p* < 0.007). Canines and lateral incisors achieved better masking (*p* < 0.001), and incisal opacities had superior outcomes (*p* < 0.019). Additionally, younger patients experienced a greater reduction (*p* < 0.026). **Conclusions**: Resin infiltration enhances the esthetics of anterior teeth with MIH in pediatric patients. While no single predictive pattern was identified, white opacities achieved greater luminance reduction and better integration with sound enamel. Factors such as age, tooth type, opacity location, lesion extent, and color significantly influence treatment effectiveness and esthetic perception.

## 1. Introduction

Hypomineralization of the first permanent molars, often also involving the incisors, was first described by Koch (1987) [1] under the term idiopathic enamel hypomineralization. Later, Weerheijm in 2001 [2] introduced the term molar–incisor hypomineralization (MIH) to define this specific developmental condition.

MIH is a qualitative enamel defect caused by disturbances during mineralization, although its clinical signs only become visible when permanent molars erupt [3]. Its etiology is multifactorial, encompassing environmental, genetic, and systemic factors, which complicates both prevention and management [4]. The diagnostic criteria for MIH were subsequently standardized by the European Academy of Paediatric Dentistry (EAPD) in 2003 [5].

Opacities extend through the full enamel thickness and present a variable clinical appearance [6]. Clinically, they present a highly variable appearance, with shades ranging from white to yellow or brown. These lesions are typically well demarcated, with clearly defined margins, and are frequently associated with dental hypersensitivity and structural weakness of the enamel, which increases the risk of post-eruptive breakdown, enables caries development, and compromises the adhesion of conventional composite resin restorations [7].

Buccal opacities on incisors have a strong esthetic impact, often prompting early treatment requests. This often leads to early treatment demand, posing a dilemma for clinicians: whether to delay intervention or to opt for conventional treatments that involve removing a large portion or even all the affected enamel. The wide variability in lesion severity, both among patients and within the same individual, has prompted the development of diverse therapeutic approaches, ranging from minimally invasive techniques to conventional restorations [8].

In this context, resin infiltration has emerged as a conservative alternative that preserves tooth structure and immediately enhances anterior esthetics [9]. This technique involves the application of a low-viscosity resin that penetrates the porous structure of hypomineralized enamel, reducing color contrast and improving surface translucency [10]. However, despite the positive aesthetic outcomes, variability in patient response and the long-term stability of the treatment remain topics of discussion [11]. Moreover, current evidence suggests that although resin infiltration provides significant esthetic improvement, its durability and effectiveness may be influenced by individual patient factors and operator technique [12].

This study aims to analyze the baseline characteristics of MIH enamel defects and to determine if there are specific patterns related to these defects that may predict clinical outcomes.

## 2. Materials and Methods

**Figure 1:**
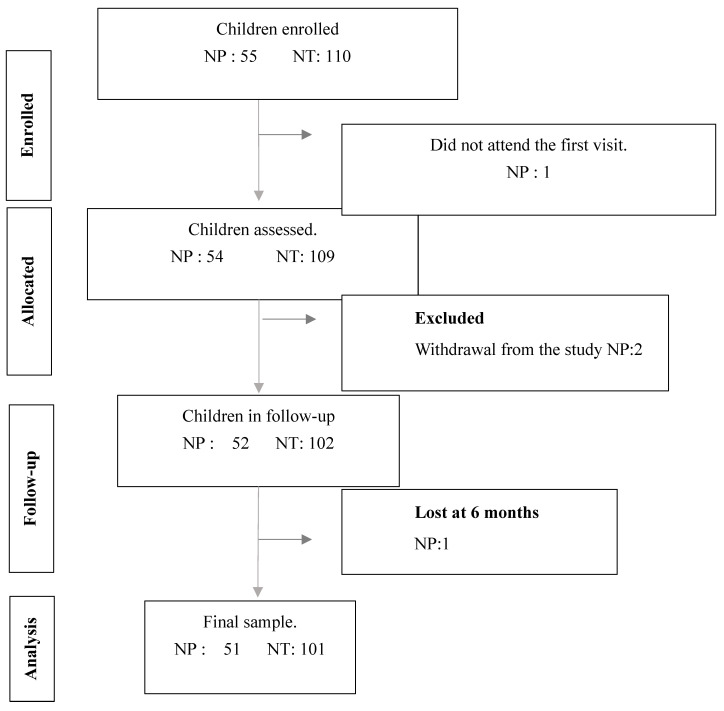
TREND flow diagram. Abbreviations. NP: number of patients; NT: number of teeth.

The study was approved by the Human Research Ethics Committee of the Experimental Research Ethics Commission of the University of Valencia (procedure number H1372162226937) and was registered in ClinicalTrials.gov within the Protocol Registration and Results System (PRS) under the identifier O00017836e2200003229. The clinical trial was registered on 25 October 2022. The study was designed as a single-arm prospective observational clinical study, as no untreated control group was included. The study followed the TREND guidelines (Figure 1).

Participants’ selection. Children aged 9 to 16 years diagnosed with molar-incisor hypomineralization (MIH) and presenting at least one opacity in the permanent anterior teeth were selected. The inclusion criteria included patients with no history of bleaching treatments or systemic conditions that could affect enamel structure. Additionally, good cooperation during the clinical procedure was required, as assessed using the Frankl behavior rating scale. The patient enrollment period began on 19 September 2023, and ended on 12 September 2024.

Sample size of the study. The sample size was calculated based on the study by Giray et al. [13] on resin infiltration in white spot lesions, considering an alpha risk of 0.05 and a beta risk of 0.2. A sample of 101 teeth with enamel defects was estimated, accounting for a 15% dropout rate. The calculation was performed using the GRANMO v7.11 Online software. All patients were included in a single group for infiltration and follow-up.

Data collection. At the first visit, after obtaining written informed consent, an initial clinical evaluation was carried out to confirm the presence of molar-incisor hypomineralization (MIH). The overall severity of the condition was assessed using the MIH Severity Index proposed by Chawla et al. [14], considering the degree of involvement of the first permanent molars.

Interventions. The resin infiltration protocol was conducted in a controlled clinical environment (Table 1). The procedure followed the recommendations of Athayde et al. [15] and Algahwe et al. [16] for ICON^®^ (DMG, Hamburg, Germany), resin infiltration, which included absolute isolation in all cases, three applications of a 15% hydrochloric acid etching gel, followed by thorough rinsing, drying, and the application of 99% ethanol. A low-viscosity resin was then applied for 30 min and light-cured. All procedures were performed by the same investigator to ensure standardization.

Data Recording. Standardized photographic records were obtained (Canon 600D camera, equipped with a 60 mm macro lens, Meike MK-14EXT ring flash, and circular diffuser. The camera was configured at F22 aperture, 1/125 s shutter speed, and ISO 100), including conventional, transillumination, and polarized images (Polar-Eyes). Color measurements were performed objectively using a spectrophotometer (Easyshade V, Vita Zahnfabrik, Germany). Individual silicone positioners (Elite Glass^®^; Zhermack, Badia Polesine, Italy) were fabricated for each patient to stabilize the device probe and to position it at the center of the opacity.

All photographic images were stored in JPEG format and processed using GIMP 2.10 for Mac (Berkeley, CA, USA) to conduct a quantitative color analysis based on the CIELAB system established by the International Commission on Illumination. The absolute values of lightness (L*), red–green coordinate (a*), and yellow–blue coordinate (b*) of each opacity were measured before and after treatment. The overall color variation (ΔE) was subsequently determined using the following formula:

ΔE = √((ΔL)^2^ + (Δa)^2^ + (Δb)^2^)

Both measurement methods were intentionally used because they provide complementary information. Spectrophotometry yields objective point-based CIELAB values under standardized illumination, while image-based analysis captures lesion morphology, color and relative surface distribution. Minor discrepancies between the two techniques may occur due to differences in measurement geometry, enamel curvature, and light interaction. These potential variations were taken into account during interpretation, and consistent trends across both methods strengthened the reliability of the results.

Lesion size was assessed from the same set of standardized photographs. The examiner outlined each opacity at baseline (T0) and post-treatment (T6) using the free selection tool in GIMP. The area of the lesion was calculated as a percentage of the total labial surface, ensuring standardized comparison between time points. All assessments were performed by a single calibrated examiner to minimize measurement variability and bias.

The aesthetic appearance of the teeth before and after treatment was evaluated using the color matching criteria of the Fédération Dentaire Internationale (FDI) [17]. Color variation was assessed at each follow-up visit. According to this scale, a restoration may range from clinically excellent or very good, showing a perfect color match with no noticeable difference in shade or translucency, to clinically good, where only minor deviations are present. A clinically adequate or satisfactory rating indicates a visible but acceptable difference that does not affect the overall aesthetics. In contrast, clinically unsatisfactory restorations display a noticeable mismatch that compromises appearance, while clinically poor cases present unacceptable color differences that require replacement.

Baseline Characteristics Analysis. A descriptive baseline assessment of the lesions was performed, recording their extent (total affected area and dental thirds), color (white yellow or brown), and visual changes after infiltration. The location of defects within the tooth and the type of affected teeth (incisors, laterals, and/or canines) were also identified.

All data regarding color, size, and location were compiled in an Excel spreadsheet for subsequent analysis.

Statistical Analysis. Descriptive statistics were used to summarize patient and tooth variables. Treatment success was evaluated based on two primary outcomes: the reduction in lesion size and the change in color, expressed as a decrease in contrast between the lesion and adjacent sound enamel.

For this study, clinical success was operationally defined as the simultaneous improvement in all objective parameters measured: reduction of ΔE, indicating decreased chromatic contrast; reduction of L, reflecting decreased luminance of the opacity; and reduction in the relative surface area of the lesion. These outcomes were assessed at the six-month follow-up visit.

Longitudinal changes in the CIELAB color coordinates (L*, a*, b*) were analyzed using linear regression models with generalized estimating equations (GEE) to account for within-patient correlations. The ΔE parameter, representing the overall color difference between the lesion and the surrounding enamel, was also analyzed using GEE models with an intercept-only structure, as time was not included as a variable.

Associations between baseline lesion characteristics (color, size, and location) and post-treatment outcomes were examined using the Wald Chi-square test for GEE models.

To identify predictors of treatment success, defined as significant changes in lesion size, L*, and ΔE values, simple linear regression models under the GEE framework were first estimated. Variables with *p* < 0.1 were subsequently included in a fully adjusted multiple regression model to control for confounding factors and to obtain unadjusted beta coefficients representing the magnitude of each variable’s effect on clinical outcomes.

The distinction between unadjusted and fully adjusted models has now been made explicit. All baseline variables were first examined individually using unadjusted simple GEE regression models. Only those variables with a *p*-value < 0.10 were subsequently included in the fully adjusted multivariable models, which were constructed to control for potential confounders and to provide adjusted estimates. Consequently, variables that did not meet the predefined inclusion threshold appear only in the unadjusted tables and not in the final adjusted models. This methodological rationale has now been clarified to enhance transparency and interpretability

Different effect estimates were reported depending on the statistical model applied. Odds ratios (OR) were used for the logistic regression analysis evaluating categorical treatment success, as they reflect the relative likelihood of achieving improvement. In contrast, unadjusted β coefficients were used in the GEE linear regression models, which assess continuous outcomes such as L, ΔE, and surface area change. Table terminology has been standardized to clearly indicate the type of model and effect estimate used.

## 3. Results

### 3.1. Sample Characteristics

**Figure 2:**
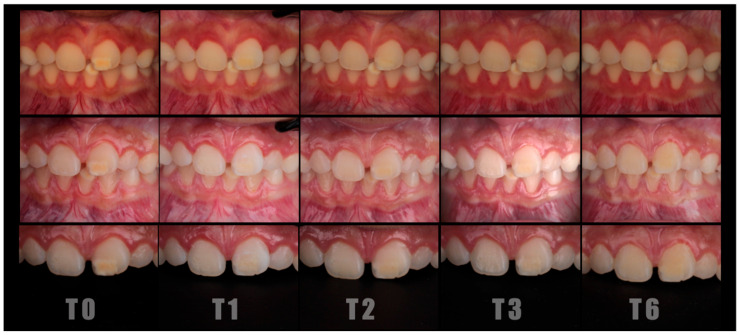
Standardized follow-up photographs at T0 (before treatment), T1 (immediately after treatment), T2 (15-day follow-up), T3 (3-month follow-up), and T6 (6-month follow-up).

The sample consisted of 101 teeth from 51 patients aged between 9 and 16 years. A higher proportion of female participants was observed. The mean Chawla severity score was 3.5 (range: 1.3–7), indicating a moderate level of MIH involvement. Most opacities were located in the maxilla (81.7%), predominantly affecting the central and lateral incisors. Regarding lesion extent, 22.6% affected less than one-third of the tooth surface, and 43.4% affected between one and two-thirds. On average, opacities covered 17.9% of the buccal surface. In terms of color distribution, 64% of lesions were white, 37% yellow, and 8% brown. Representative standardized clinical images illustrating these baseline characteristics are shown in Figure 2.

At baseline (T0), color analysis showed that MIH opacities generally exhibited a predominantly yellow hue with a slight reddish tendency. The mean luminance (L*) value was approximately 70 according to spectrophotometric measurements, while slightly lower values were obtained from image-based analysis.

Initial spectrophotometric analysis revealed significant differences among opacity color groups. White opacities showed higher L* values (*p* = 0.049), indicating greater brightness, whereas yellow and brown opacities exhibited significantly higher a* (*p* = 0.001) and b* (*p* < 0.001) values, reflecting increased reddish and yellowish components.

### 3.2. Evaluation of Color Change

**Figure 3:**
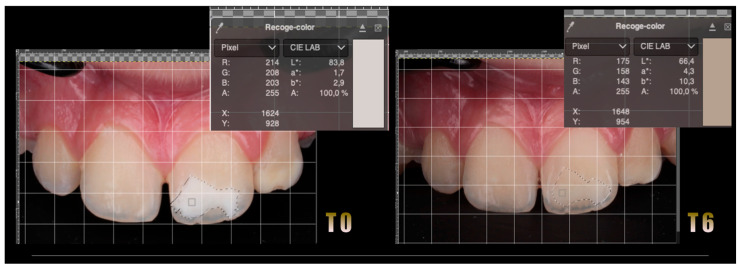
Color analysis at T0 (before treatment) and at T6 (6-month follow-up).

Following resin infiltration, a significant reduction in the color difference (ΔE) of the opacities was observed, indicating improved esthetic integration with the surrounding enamel. White opacities showed a decrease in L* values, reflecting reduced brightness and better blending, while yellow and brown opacities exhibited decreases in a* and b* values, approaching those of adjacent sound enamel. These trends are illustrated in Figure 3. However, greater variability was observed in darker opacities, suggesting that lesion type and depth influence treatment outcomes.

### 3.3. Evaluation of Relative Surface Change

**Figure 4:**
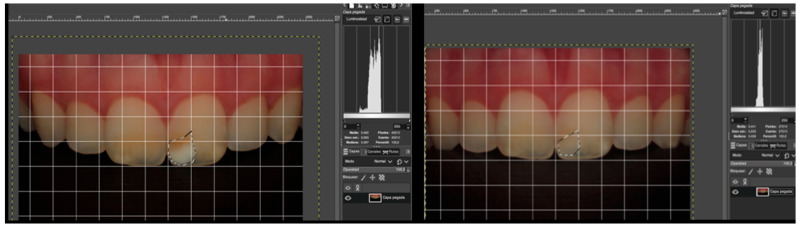
Lesion size analysis at T0 (before treatment) and at T6 (6-month follow-up). To assess the change in the size of the opacity surface at T0 and T6, the boundaries of the opacities were manually outlined using the free selection tool, obtaining their dimensions in pixels before and after resin infiltration. To facilitate the interpretation of the reduction in lesion size, the pixel values from the images were converted into percentages.

The analysis of lesion size before and after treatment revealed a significant reduction in the relative surface area occupied by opacities. White opacities showed the greatest reduction compared with yellow and brown ones (*p* = 0.029). Larger initial lesions demonstrated greater post-treatment reduction (*p* = 0.007). Patient age also affected outcomes, with older patients showing a smaller reduction in relative surface area (*p* = 0.053). These findings underscore the relevance of baseline opacity characteristics as predictors of resin infiltration success, as illustrated in Figure 4.

### 3.4. Predictive Models of Treatment Success

Multivariable regression models identified baseline color, initial lesion size, and patient age as the main predictors of infiltration efficacy (Table 2). The model assessing reduction in relative lesion area showed that white opacities experienced significantly greater decreases than yellow and brown ones (*p* = 0.028). A negative correlation was found between initial lesion size and treatment effectiveness, indicating that larger lesions achieved greater reductions post-treatment (*p* = 0.007). Age showed a consistent trend (*p* = 0.053), with each additional year associated with a 0.5% smaller reduction in lesion size.

In relation to the reduction in luminosity (L*) (Table 3), the findings highlight several positive aspects of treatment performance. Lateral incisors showed a significantly greater decrease in brightness compared with central incisors (β = –8.87; *p* = 0.001), indicating a more effective masking of opacities and, consequently, a higher esthetic improvement.

Lesions located in the incisal third demonstrated a greater reduction in luminosity than those in the middle third (β = 5.13; *p* = 0.019), suggesting that opacities in this region respond particularly well to resin infiltration.

Similarly, smaller lesions, those involving less than one-third of the surface, achieved a more pronounced improvement in brightness than larger ones (β = 5.90; *p* = 0.024), reinforcing the effectiveness of the treatment in localized defects.

A positive trend was also observed, indicating that the smaller the affected area, the greater the reduction in luminosity (β = 0.17; *p* = 0.059), further supporting that resin infiltration is especially effective for small, well-defined opacities.

Analyzing the ΔE (color difference) change (Table 4), lateral incisors (LI) and canines (C) showed greater ΔE shifts compared with central incisors (CI), with additional increases of 5.7 and 16.5 points, respectively. These results indicate a higher degree of color blending and esthetic improvement in these tooth types. Furthermore, opacities located in the incisal third presented a more pronounced ΔE change than those in the middle third (β = −3.37; *p* = 0.022), indicating that lesions positioned toward the incisal area respond particularly well to treatment.

Overall, these findings emphasize the effectiveness of resin infiltration in achieving visible color harmonization, especially in lateral incisors, canines, and incisal opacities, leading to notable aesthetic improvements after treatment.

These results suggest that the baseline characteristics of the opacities, particularly their initial color, size, play a decisive role in predicting the success of resin infiltration. More extensive or darker lesions may reflect a higher degree of subsurface porosity and internal enamel disorganization, potentially limiting resin penetration and reducing the aesthetic improvement achieved.

## 4. Discussion

Analyzing baseline opacity characteristics and post-treatment changes helped identify factors influencing esthetic improvement. In this regard, the reduction in lesion size and color modification suggested that certain baseline characteristics may influence treatment response. Previous studies have already suggested that baseline features—particularly opacity color, lesion extent, and enamel porosity—play a pivotal role in determining the response to resin infiltration. Warner et al. [18] reported that darker lesions, often associated with increased depth and altered mineral composition, show reduced masking capacity, reinforcing color as a key predictive factor. Similarly, Athayde et al. [15] demonstrated that lesion size and surface porosity significantly influence the degree of resin penetration and the magnitude of esthetic improvement, supporting the importance of considering lesion extent. Our findings are also aligned with the work of Bhandari et al. [9], who emphasized the predictive relevance of initial lesion characteristics in determining infiltration outcomes. These findings highlight color as a key predictor and support tailoring treatment to lesion type. However, further investigation is required to better understand the impact of these factors in order to optimize clinical protocols and maximize esthetic outcomes [19].

Regarding the reduction in lesion size, white opacities showed a significantly greater decrease compared with yellow and brown ones. This observation aligns with the findings of Warner et al. [18], who suggested that the poorer response of yellow and brown lesions may be associated with greater depth and altered mineral composition, which limit complete masking. Our results support the link between opacity color and the structural complexity of the defect, showing that the chromatic and structural characteristics of opacities significantly influence treatment effectiveness. These findings emphasize the predictive value of lesion color for clinical outcomes and reinforce the need for tailored therapeutic approaches according to lesion type.

Lesions with greater initial extent exhibited a larger reduction in surface area after treatment, in agreement with prior reports indicating higher infiltration effectiveness in defects with increased surface porosity [10]. Similarly, Athayde et al. [15] proposed that extensive lesions, due to their higher porosity, allow deeper resin penetration and consequently better therapeutic response. Nonetheless, other factors, such as enamel composition and structural heterogeneity, may also influence both treatment efficacy and long-term stability.

This apparent divergence between surface area reduction in larger lesions and greater L improvement in smaller lesions can be explained by the different structural and optical characteristics of MIH defects. Larger lesions often present deeper and more heterogeneous subsurface porosity, which facilitates greater resin penetration and results in a more pronounced reduction in their overall surface extent. Conversely, smaller and more superficial lesions typically exhibit more uniform mineral loss and clearer demarcation, allowing the infiltrant to homogenize light reflection more effectively and produce a stronger decrease in L. Therefore, these outcomes reflect complementary mechanisms rather than contradictory behavior, highlighting how lesion depth, extent, and porosity patterns modulate the esthetic response to resin infiltration.

In relation to color change, canines and lateral incisors showed a more pronounced reduction in L* values after resin infiltration. This suggests that treatment response may depend on anatomical and structural differences among tooth types, as well as on the morphology of the hypomineralized defects. Color measurement variability may also influence results due to differences in enamel curvature and surface reflection.

The influence of opacity position along the tooth surface was also evident: a greater decrease in L* values was observed in the incisal third, while reductions were significantly smaller in the middle third. These findings are consistent with Warner et al. [18] who attributed the greater effectiveness of infiltration in the incisal region to the disorganized arrangement of enamel prisms and lower mineralization compared with the middle third. Such structural differences may facilitate deeper resin penetration, explaining the variability in treatment response according to lesion location.

Concerning lesion extent by dental thirds, opacities affecting less than one-third of the surface showed the largest change in ΔE, indicating superior esthetic improvement in smaller, more localized defects [20,21]. To the best of our knowledge, no published data are available on this aspect.

Patient age also influenced treatment response, with a smaller reduction in lesion area observed in patients over 13 years old. This may be related to enamel maturation and its reduced permeability to resin infiltration [12]. Recent studies suggest that hypomineralized enamel undergoes partial natural remineralization over time, which could limit infiltration efficacy in older patients. This factor may explain the variability in outcomes by age, emphasizing the importance of early intervention to maximize treatment benefits [10].

The predictive models of treatment success developed in this study reinforced the importance of considering opacity baseline characteristics in treatment planning. In line with the findings of Bhandari et al. [9] our multivariable regression analysis demonstrated that the initial hue and lesion extent were the main determinants of color improvement and surface reduction. Furthermore, cross-validation confirmed a moderate reliability of the predictive model suggesting, according to Rodd et al. [22], the need for additional studies to explore other variables influencing clinical outcomes.

Clinically, these results emphasize the need for a thorough pre-treatment assessment to predict esthetic outcomes. In daily practice, clinicians should consider factors such as opacity color, extent, and location, as well as patient age, when planning treatment with resin infiltration. White opacities in younger patients tend to yield the most favorable aesthetic outcomes, whereas darker or more extensive lesions may require combined or alternative therapeutic approaches.

Recent clinical evidence supports the use of resin infiltration as an effective, minimally invasive, and tissue-preserving approach for the esthetic management of anterior MIH lesions in pediatric patients. In a six-month clinical evaluation, Altan and Yilmaz [23] demonstrated that this technique significantly improved the color and appearance of hypomineralised enamel, achieving stable outcomes over time. However, clinicians should set realistic expectations, particularly for yellow or brown opacities, where complete masking may be difficult to achieve. 

This study has several limitations. The sample size and six-month follow-up period restrict the generalizability of the findings and do not allow assessment of long-term color stability. In addition, the use of both spectrophotometric and image-based methods may introduce minor measurement variability. The absence of an untreated control group limits comparison with the natural progression of MIH opacities. Another methodological limitation concerns the use of JPEG files for photographic acquisition. JPEG is a compressed, non-linear, and device-dependent format that is not ideal for extracting precise CIELAB values. Although this constraint was mitigated by strict standardization of the photographic protocol and by complementing image-based measurements with spectrophotometric data, the use of RAW files would have allowed more robust and distortion-free color analysis. Future studies should incorporate RAW imaging to enhance the accuracy of digital color measurements.

A major limitation of this study is its 6-month follow-up period, which is too short to evaluate the long-term stability of resin infiltration. Several authors have highlighted that TEGDMA-based infiltrants may undergo optical aging, progressive yellowing, or changes in refractive index over time, potentially reducing the masking effect achieved initially [10,12]. Warner et al. [18] and other clinical studies have also suggested that deeper or structurally complex lesions may experience color rebound or partial loss of masking as enamel undergoes natural aging and dehydration–rehydration cycles. Because our study did not extend beyond 6 months, it was not possible to assess infiltrant stability, color durability, or potential degradation of the resin within enamel porosities. Longer longitudinal studies are therefore required to determine whether the esthetic improvements observed in the short term remain stable over time.

Finally, unmeasured factors such as enamel hydration, oral hygiene, or potential optical aging of the infiltrant may also influence outcomes. Future studies with larger samples and extended follow-up are required to validate these observations.

## 5. Conclusions

Although no specific response pattern of MIH lesions to resin infiltration could be established, the results of this study demonstrated that white opacities tend to exhibit a marked reduction in luminosity, achieving greater blending with the surrounding healthy enamel compared with yellow or brown opacities.

Age, tooth type, arch location (maxillary or mandibular), opacity position within the tooth (incisal or middle third), lesion extent, and color were all significantly associated with both objective improvement and enhanced subjective perception of esthetic outcomes following treatment.

## Figures and Tables

**Table 1:** Resin Infiltration Protocol.

Step	Procedure	Details
Preparation	Absolute isolation	Using a rubber dam to ensure a moisture-free environment.
Etching	Application of 15% hydrochloric acid	Duration: 2 min.
Rinsing	Aspiration and water rinsing	Duration: 30 s.
Drying	Cleaning and drying with ethanol	Application of 99% ethanol.
Infiltration	Application of low-viscosity TEGDMA-based resin (ICON^®^, DMG)	Duration: 30 min. Prolonged application time allows deep penetration into the porous enamel structure.
Polymerization	Light curing	Initial curing for 40 s, application of glycerin to prevent the oxygen-inhibition layer, followed by an additional 40 s of curing.
Polishing	Removal of resin excess and surface finishing	Polishing with Eve™ discs.

**Table 2:** Logistic Regression Analysis of Factors Associated with Treatment Success.

Variable	OR	95% CI	*p*-Value
Age (years)	0.99	0.71–1.37	0.667
Sex			
Boy	1		
Girl	0.52	0.12–2.26	0.381
Parental relationship			0.779
Mother	1		
Father	0.62	0.13–2.97	0.549
Other	1.33	0.12–14.6	0.818
Tooth type			0.299
Central incisor (CI)	1		
Lateral incisor (LI)	0.56	0.26–1.20	0.133
Canine (C)	0.40	0.04–4.09	0.437
Arch			
Maxillary	1		
Mandibular	0.26	0.08–0.88	0.030 *
Opacity location			
Incisal third	1		
Middle third	0.93	0.27–3.25	0.910
Color			0.032 *
White	1		
Yellow	0.33	0.09–1.17	0.085
Brown	0.16	0.03–0.74	0.019 *
Extension			0.052
<1/3 of surface	1		
1/3–2/3	0.06	0.01–0.60	0.016 *
>2/3	0.13	0.01–1.47	0.099
% Surface area affected	1.03	0.99–1.07	0.192

OR = Odds Ratio Effect estimates expressed as odds ratios (OR); CI = Confidence Interval. * *p* < 0.05 indicates statistical significance.

**Table 3:** Factors Associated with Luminosity (L*) Reduction After Resin Infiltration.

Variable	β (Unadjusted)	95% CI	*p*-Value
Tooth type			
Central incisor	—	—	—
Lateral incisor	−8.87	−14.02–−3.72	0.001 *
Opacity location			
Incisal third	—	—	—
Middle third	5.13	0.83–9.44	0.019 *
Lesion extent			
<1/3 of surface	—	—	—
>2/3 of surface	5.90	0.79–11.01	0.024 *
% Surface area occupied	0.17	−0.01–0.35	0.059

β = unadjusted regression coefficient; CI = confidence interval. * *p* < 0.05 indicates statistical significance. Linear GEE Regression: Unadjusted β Coefficients for Factors Associated with (L*) Reduction.

**Table 4:** Factors Associated with ΔE (Color Difference) Change After Resin Infiltration.

Variable	β (Unadjusted)	95% CI	*p*-Value
Age (years)	0.78	0.09–1.46	0.026 *
Sex			
Boy	—	—	—
Girl	0.81	–2.80–4.42	0.659
Tooth type			< 0.001 ***
Central incisor (CI)	—	—	—
Lateral incisor (LI)	5.73	1.63–9.83	0.006 **
Canine (C)	16.50	8.48–24.50	< 0.001 ***
Arch			
Maxillary	—	—	—
Mandibular	2.98	–2.41–8.37	0.279
Opacity location			
Incisal third	—	—	—
Middle third	–3.37	–6.25––0.48	0.022 *
Color			0.213
White	—	—	—
Yellow	2.15	–1.56–5.86	0.256
Brown	6.86	–2.28–16.00	0.141
Lesion extent			0.753
<1/3 of surface	—	—	—
1/3–2/3	1.63	–2.62–5.88	0.452
>2/3	0.56	–3.27–4.39	0.773
% Surface area occupied	0.01	–0.09–0.12	0.834

β = unadjusted regression coefficient; CI = confidence interval. * *p* < 0.05, ** *p* < 0.01, *** *p* < 0.001 indicate statistical significance. Linear GEE Regression: Unadjusted β Coefficients for Factors Associated with ΔE Change.

## Data Availability

The data will be made available upon reasonable request to the authors.

## Abbreviations

The following abbreviations are used in this manuscript:

EAPD	European Academy of Paediatric Dentistry
MIH	Molar incisor hypomineralization
UV	University of Valencia
PRS	Protocol Registration and Results System
GEE	Generalized estimating equations
CI	Central incisor
LI	Lateral incisor
C	Canine
OR	Odds Ratio
CI	Confidence Interval
